# Maternal Prepregnancy 5-Hydroxytryptamine Exposure Affects the Early Development of the Fetus

**DOI:** 10.3389/fphys.2022.761357

**Published:** 2022-03-17

**Authors:** Yu Han, Meng Zhang, Jiahui Duan, Leyi Li, Jinge Du, Hui Cheng, Sheng Zhang, Yanhui Zhai, Xinglan An, Qi Li, Xueming Zhang, Ziyi Li, Bo Tang

**Affiliations:** ^1^College of Veterinary Medicine, Jilin University, Changchun, China; ^2^Academy of Translational Medicine, First Hospital, Jilin University, Changchun, China

**Keywords:** 5-HT, maternal effect, placenta, fetus, prepregnancy

## Abstract

In recent decades, the increasing incidence of depression has contributed to an increase in the use of serotonergic drugs, such as antidepressants, which predisposes humans to serotonin syndrome. Serotonin syndrome is caused by elevated serotonin levels in the central and peripheral nervous systems. It has been well documented that the development of offspring can be affected by maternal exposure to environmental challenges, such as stress, diseases, or an unhealthy diet during pregnancy. Serotonin, also called 5-hydroxytryptamine (5-HT), is widely expressed in the female reproductive system and plays an important role in the development of follicles and embryos. However, whether the suffering of the mother from serotonin syndrome before pregnancy affects fetal development is still uncertain. In the present study, to explore the effect of maternal prepregnancy 5-HT exposure on the fetus, intraperitoneal injection of 5-HT was used to change maternal prepregnancy 5-HT levels. It was found that maternal prepregnancy 5-HT exposure significantly reduced the body weight and liver weight and the levels of estrogen and progesterone in female mice. Although there was no significant difference in the cleavage rate and blastocyst rate between the 5-HT and control groups, maternal prepregnancy 5-HT exposure increased the percentage of embryo resorption, decreased placental weight, and led to placental inflammation at E13.5. Notably, 5-HT exposure caused weight loss in the offspring at 2 weeks. These results suggested that maternal prepregnancy 5-HT exposure could affect the development of the offspring, which was partly caused by reduced hormonal secretion and placental inflammation.

## Introduction

During the critical period of development, adverse environmental exposure, such as stress, toxins, or an unhealthy diet, will affect the growth trajectory before birth, significantly impact health, and increase disease susceptibility in offspring (Han et al., 2018). Maternal factors play important roles in offspring development and postnatal life in adulthood. In mice, exposure to maternal obesity before and during pregnancy can cause early embryonic developmental delay and fetal developmental delay (Jungheim et al., 2010; Luzzo et al., 2012; McPherson et al., 2015; Sasson et al., 2015; Wu et al., 2015). It has been reported that maternal iron limitation mediated by hepcidin during pregnancy causes embryonic anemia, tissue iron deficiency, and weight loss (Sangkhae et al., 2020). Offspring growth restriction and impaired metabolism in adulthood result from hyperglycemia exposure before and during pregnancy (Bianco and Josefson, 2019). The transgenerational inheritance of epiphenotypes in mammals could be caused by disruptions to gut microbiota, DNA methylation in oocytes, and other pathways (Chen et al., 2016; Li et al., 2016; Consitt et al., 2018; Han et al., 2018; Zhang et al., 2018). Maternal obesity during pregnancy can cause cognitive and social behavior dysfunction in offspring (Liu et al., 2021). These deficiencies derive from changes in the structure of the gut microbiota and alterations in metabolites in both the mother and offspring (Liu et al., 2021).

5-Hydroxytryptamine (5-HT), also known as serotonin, is a neurotransmitter that is widely distributed in the central nervous system and gastrointestinal tract, where it also functions as a hormone and a growth factor. In humans, most 5-HT is found in peripheral tissues, of which approximately 90% is synthesized and mainly distributed in intestinal enterochromaffin cells (Kato, 2013). Notably, previous evidence has shown that 5-HT is detected in reproductive tissues and plays an essential role in cell proliferation, follicular development, embryo development, and fetal growth (Tanaka et al., 1993; Edwards, 1995; Il'kova et al., 2004; Amireault and Dube, 2005; Kim et al., 2010; Oberlander, 2012; Amireault et al., 2013). 5-HT is involved in the pathophysiology of many mental illnesses and is the target of many drug therapies, such as selective serotonin reuptake inhibitor (SSRI) drugs widely used in depression and anxiety (Brummelte et al., 2017). In humans, ingestion of a combination of two or more serotonin (5-HT)-enhancing drugs and overdose of a single drug can cause serious adverse reactions, such as serotonin syndrome (Haberzettl et al., 2015). Serotonin syndrome is a potentially life-threatening drug-induced condition caused by too much serotonin in the brain synapses (Foong et al., 2018). The incidence of serotonin syndrome has gradually increased worldwide, which has mostly resulted from the use of antidepressants, such as monoamine oxidase inhibitors, selective serotonin reuptake inhibitors, and serotonin-norepinephrine reuptake inhibitors (Wang et al., 2016b; Bartlett, 2017). Serotonin syndrome usually manifests as a triad of autonomic dysfunction, neuromuscular excitement, and altered mental status, although not all of these symptoms consistently exist in all patients with the disorder (Boyer and Shannon, 2005). However, there is no evidence of whether a mother suffering from serotonin syndrome will impact the development of the fetus.

As a place for the exchange of nutrients and metabolic waste between the mother and the fetus, the placenta plays an important role in maintaining pregnancy by secreting a variety of hormones, growth factors, and cytokines (Cross, 1998; Rossant and Cross, 2001). Once the development and function of the placenta are impaired, the nutrients, hormones, and growth factors required for embryonic and fetal development cannot be guaranteed (Rossant and Cross, 2001; Regnault et al., 2002). It is easier to transfer toxic maternal substances to the fetus through the placental barrier, which will inevitably damage the growth and development of the embryo and fetus (Dimasuay et al., 2016). In addition, the placenta is an important barrier for fetal immune protection during pregnancy and can protect the fetus from maternal immune system attack (Regnault et al., 2002). Prior evidence has shown that placental developmental disorders can hinder the normal growth of the fetus, causing miscarriage, fetal growth restriction, premature delivery, and stillbirth (Rock et al., 2020). During pregnancy, placental inflammation and impaired function caused by maternal metabolic endotoxemia lead to an adverse early-life environment, which is connected to metabolic dysfunction in offspring (Elahi et al., 2009). It has been reported that maternal circulating microRNAs (miRNAs) can cross the placenta and affect the phenotype of offspring (Nelson and Nadeau, 2010; Sun et al., 2020). Cote found that there were the same 5-HT levels in both t*ph1*^+/−^ (tryptophan hydroxylase and the rate-limiting enzyme in 5-HT synthesis) female mice and wild-type females, but in *tph1*^−/−^ females, it dropped to 3–15% of the level of wild-type females (Cote et al., 2003). Interestingly, the offspring derived from heterozygous mothers have been exposed to a normal 5-HT level, no matter whether their genotype is *tph1*^−/−^ or *tph1*^+/−^(Francine et al., 2006). Therefore, maternal 5-HT levels are essential for the normal development of fetuses.

However, it is unclear whether the suffering of mothers from serotonin syndrome before pregnancy can affect the development of offspring. Therefore, we used intraperitoneal injection to change prepregnancy 5-HT levels to explore the effects of maternal prepregnancy 5-HT exposure on the mother, placenta, and fetus. This study provides new insights into the relationships among 5-HT, mother, and fetus and develops new directions for the effects of 5-HT on intergenerational inheritance.

## Materials and Methods

### Animals and Treatment

All animal treatments in this study were performed in strict accordance with the guidelines of the Animal Welfare Research Ethics Committee of Jilin University. ICR mice were purchased from Liaoning Changsheng Biotechnology Co., Ltd. (Liaoning, China), fed regular chow, and housed in ventilated cages. Female wild-type mice (5 weeks old) were randomly divided into two groups (*n* = 6): the control group and the 5-HT group. The control group was intraperitoneally injected with saline solution, the 5-HT group was intraperitoneally injected with 100 mg/kg/day 5-HT (serotonin creatinine sulfate, Sigma, United States) solution for 10 days, and the body weight was recorded every 5 days.

**Figure 1:**
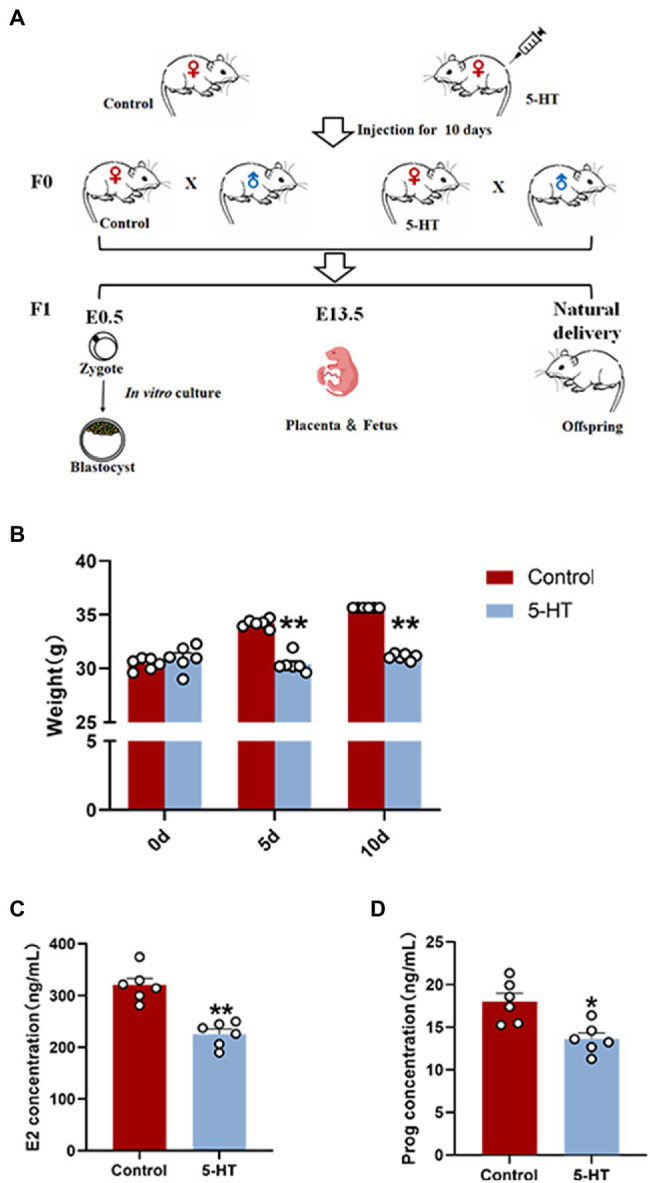
Effects of maternal prepregnancy 5-HT exposure on female mice. **(A)** Schematic of the maternal 5-HT exposure and breeding; **(B)** the changes in maternal body weight on the 5th and 10th day of 5-HT exposure; **(C)** the maternal levels of estradiol (E2); and **(D)** progesterone (Prog) after maternal prepregnancy 5-HT exposure. *n* = 6, ^*^p < 0.05, and ^**^p < 0.01.

After maternal prepregnancy 5-HT exposure, female mice, as well as the females in the control group, were mated overnight with males of proven fertility. According to the presence of a vaginal plug, gestational age was determined. Thus, Day 0 was defined as midnight of the mating day. Next, pregnant mice were divided into three groups, which were used for culturing zygotes *in vitro*, collecting fetuses and placentas, and obtaining offspring from natural delivery. On E0.5, some of the pregnant mice were sacrificed by cervical dislocation to obtain zygotes, and then, the zygotes were cultured *in vitro*. On E13.5, female mice were euthanized by carbon dioxide asphyxiation, and then, we removed the uterus to obtain fetuses and placentas. The rest of the female mice experienced natural delivery to obtain offspring, and we recorded the weights of the offspring at 2 weeks of age. The detailed experimental process is shown in Figure 1A.

### Preimplantation Embryo Collection and Culture

After the injection of 5-HT for 10 days, female mice were injected with 5 U of pregnant mare serum gonadotropin to achieve superovulation, followed by the injection of 5 U of human chorionic gonadotropin (HCG; Cen’s, Hangzhou, China) 48 h after pregnant mare serum gonadotropin (PMSG; Shusheng, Ningbo, China) priming. Subsequently, female mice were mated overnight with males of proven fertility. According to the presence of a vaginal plug, gestational age was determined. Thus, Day 0 was defined as midnight of the mating day. Zygotes were isolated from oviduct ampullae and then cultured in KSOM medium (Sigma, United States) at 37°C after removing granulosa cells by hyaluronidase.

### Placenta and Fetus Collection

On E13.5, female mice were euthanized by carbon dioxide asphyxiation, and the structure and pathological changes were dissected and examined. The uterus was removed to obtain the placenta and fetuses for inspection and data recording.

### Hematoxylin and Eosin Staining

To detect histopathological changes, placental tissues were collected and fixed in 4% paraformaldehyde fixing solution for 48 h, dehydrated in a graded ethanol series, embedded in paraffin wax, and cut into 5-μm-thick sections. The sections were stained with hematoxylin and eosin (H&E). Finally, pathological changes in placental tissues were observed under a light microscope.

### ELISA

Orbital blood was collected from female mice after 10 day injection of 5-HT, as well as the pregnant at E13.5. The orbital blood was centrifuged at 3,000 rpm at 4°C for 10 min for subsequent experiments. The levels of estrogen and progesterone in the mice were measured using commercially available estrogen and progesterone ELISA kits (Nanjing Jiancheng Bioengineering Institute, Nanjing, China) according to the instructions of the manufacturer. All samples were measured in triplicate in a single assay.

### RNA Extraction and Reverse Transcription

RNAiso plus (Beyotime, Shanghai, China) was used to extract total RNA from tissues following the manufacturer’s instructions. Subsequent reverse transcription was performed using the First-Strand cDNA Synthesis kit (Promega, Madison, WI, United States).

### Fluorescent Quantitative PCR

The primers used in the following quantitative PCR (qPCR) are listed in Table 1. The qPCR mixture consisted of 10 μl of SYBR green premix, 1 μl of each forward and reverse primer, 1 μl of cDNA, and 7 μl of RNase-free water. The next treatment was denaturation at 95°C for 180 s and then amplification with 40 cycles of 95°C for 5 s and 60°C for 30 s, followed by melting curve analysis (95°C for 60 s, 60°C for 60 s, and 95°C for 1 s) and finally cooling at 4°C. The data were obtained from at least three repeats and calculated to obtain the relative transcription level using the 2^−△△CT^ method.

**Table 1:** Primers used in qPCR.

Primer	Forward	Reverse
VEGFA	GGAGAGACTTCGAGGAGCACTT	GGCGATTTAGCAGCAGATATAAGAA
ANGPT2	TTAGCACAAAGGATTCGGACAAT	TTTTGTGGGTAGTACTGTCCATTC
HAND1	GGCAGCTACGCACATCATCA	CCTGGCATCGGGACCATAG
ESX1	TTTTCCAGCGCGTCCAGTA	TCGGGCAAGCTCCACTCT
BCL2	ATGCCTTTGTGGAACTATATGGC	GGTATGCACCCAGAGTGATGC
IL-8	CGGCAATGAAGCTTCTGTAT	CCTTGAAACTCTTTGCCTCA
IL-6	CCAGAGATACAAAGAAATGATGG	ACTCCAGAAGACCAGAGGAAAT
IL-1β	CAAGCAATACCCAAAGAAGAAGA	ATTAGAAACAGTCCAGCCCATAC
TNF-α	CGGAGTCCGGGCAGGTCTACTTT	GTCCAGGTCACTGTCCCAGCATC
*β*-Actin	GACGGCCAGGTCATCACTATTG	AGGAAGGCTGGAAAAGAGCC

### Statistical Analysis

The analysis of data and graphs was performed with GraphPad Prism 8.0 software. Data were analyzed using a t-test or one-way ANOVA. The post-hoc test to obtain *p* values was the Holm-Sidak method. All data are presented as the mean ± S.E.M, with a value of *p* < 0.05 considered to be statistically significant.

## Results

### Effects of Maternal Prepregnancy 5-HT Exposure on Female Mice

Here, we first examined the effect of 5-HT exposure on female mice by weighing at 0 d, 5 d, and 10 d during the intraperitoneal injection of 5-HT. The results showed that during the 10-day-injection period, the weights of female mice in the control group were increased, while the weight of the mice in the 5-HT group did not change. On Days 5 and 10 after intraperitoneal injection of 5-HT, the weights of females in the 5-HT group were significantly lower than those in the control group (*p* < 0.01; Figure 1B). The results revealed that 5-HT exposure prevented weight gain in mice.

To preliminarily investigate the correlation between 5-HT and female reproduction, the hormonal levels of blood from female mice in the presence or absence of 5-HT exposure were detected. The results showed that 5-HT exposure led to a marked reduction (~30%) in estrogen levels and progesterone levels, confirming the negative effect of 5-HT on female mice (Figures 1C,D).

### Effects of Maternal Prepregnancy 5-HT Exposure on Pregnant Mice

**Figure 2:**
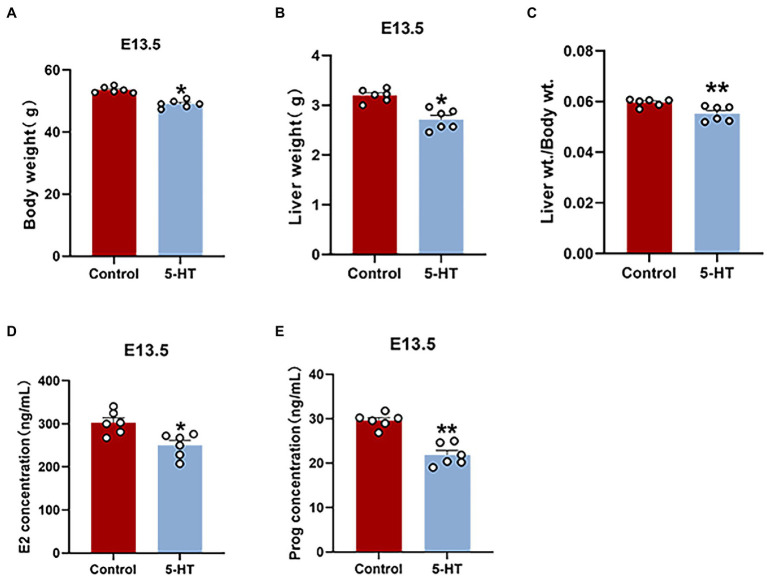
Effects of maternal prepregnancy 5-HT exposure on pregnant mice. After maternal prepregnancy 5-HT exposure, female mice were mated overnight with males of proven fertility. On E13.5, the maternal phenotype was detected. **(A)** The changes in maternal body weight and **(B)** liver weight at E13.5 after maternal prepregnancy 5-HT exposure; **(C)** the liver/body weight ratios after maternal prepregnancy 5-HT exposure; **(D)** the maternal levels of estradiol (E2); and **(E)** progesterone (Prog) at E13.5 after maternal prepregnancy 5-HT exposure. *n* = 6, ^*^p < 0.05, and ^**^p < 0.01.

To investigate the effect of 5-HT on pregnant mice, we weighed mice and collected tissues to perform data analysis on E13.5. Notably, female mice derived from the 5-HT group had a much lower pregnancy weight and liver weight at E13.5 than the controls (*p* < 0.05; Figures 2A,B), which was consistent with our above results. There was also a remarkable reduction in the liver/body weight ratios in the 5-HT group compared with the control group (*p* < 0.01; Figure 2C). Additionally, concordant with the lower hormone levels in nonpregnant mice, estrogen and progesterone levels were obviously reduced in pregnant mice exposed to 5-HT (Figures 2D,E). Taken together, 5-HT treatment reduced the body weight and hormone levels in both nonpregnant and pregnant mice.

### Effect of Maternal Prepregnancy 5-HT Exposure on the Placenta

**Figure 3:**
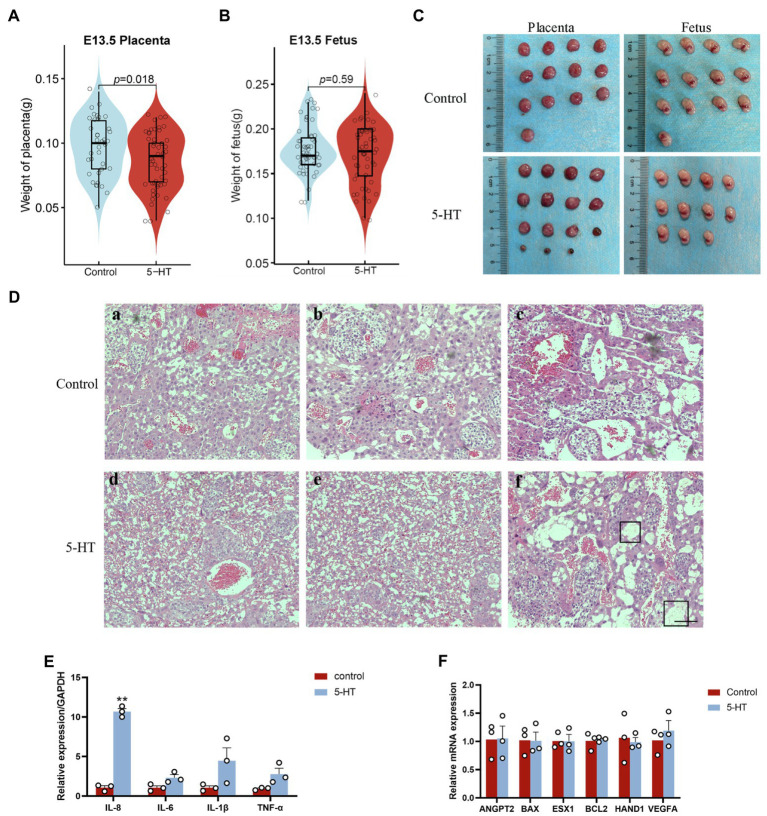
Effects of maternal prepregnancy 5-HT exposure on the placenta. On E13.5, female mice were euthanized by carbon dioxide asphyxiation, and the uterus was removed to obtain the placenta and fetus. **(A)** The changes in placental weight and **(B)** fetal weight at E13.5 after maternal prepregnancy 5-HT exposure; **(C)** representative images of placentas and fetuses at E13.5 after maternal prepregnancy 5-HT exposure; and **(D)** histology of placentas after maternal prepregnancy 5-HT exposure. The black boxed area indicates inflammatory cell infiltration and edema. Scale bar, 100 μm. **(E)** The mRNA expression levels of the key genes in placental development, angiogenesis, and apoptosis after maternal prepregnancy 5-HT exposure. **(F)** The mRNA abundance of inflammatory factors after maternal prepregnancy 5-HT exposure. *n* = 6, ^*^p < 0.05, and ^**^p < 0.01.

Next, we explored the effect of 5-HT on the placenta at E13.5. Notably, placental weight in the 5-HT group was markedly decreased compared to that in the control group (Figure 3A), while fetal weight showed no significant difference in all groups (Figure 3B).

H&E staining and qPCR were used to better understand the detailed effects of 5-HT on placental development. The H&E staining results indicated that the placentas from the 5-HT group had relatively more red blood cells (Figure 3D). There was local inflammatory cell infiltration and edema in placental tissue after maternal prepregnancy 5-HT exposure (Figure 3D). Consistently, our qPCR data showed that the mRNA levels of the proinflammatory cytokine IL-8 were obviously increased after 5-HT injection (*p* < 0.01; Figure 3E). Although the expression of IL-6, IL-1β, and TNF-α was not significantly different, it showed an increasing tendency (Figure 3E). These results revealed that 5-HT exposure led to placental inflammation. However, 5-HT exposure did not affect the mRNA expression of the angiogenesis-related genes VEGFA and ANGPT1, apoptosis-related genes BAX and BCL2, or development-related genes ESX1 and HAND1 (Figure 3F).

### Effects of Maternal Prepregnancy 5-HT Exposure on Preimplantation Embryo Development

**Figure 4:**
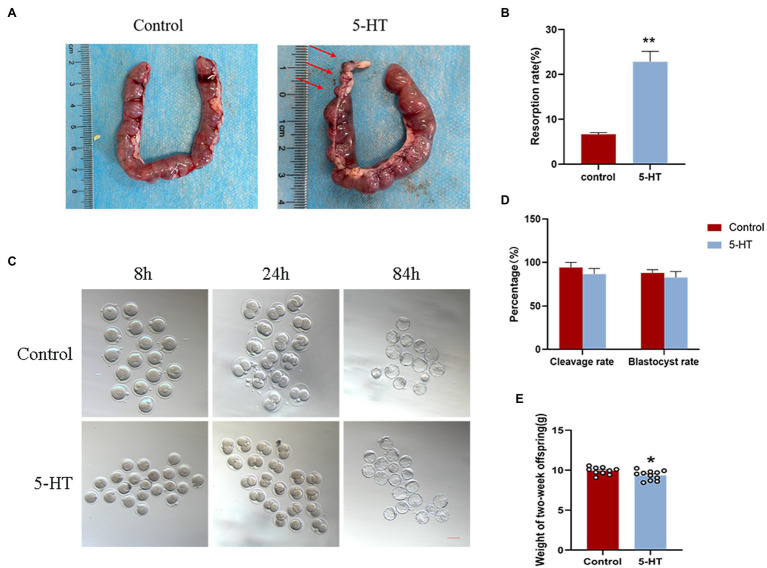
Effect of maternal prepregnancy 5-HT exposure on embryos, fetuses, and offspring. After maternal prepregnancy 5-HT exposure, female mice were mated overnight with males of proven fertility. On E0.5, some of the pregnant mice were sacrificed by cervical dislocation for zygote collections, and then, the zygotes were cultured *in vitro*. The other pregnant mice were naturally delivered to obtain offspring. **(A)** Representative images of different stages during embryonic development after maternal prepregnancy 5-HT exposure (8 h, 24 h, and 84 h after mating). Scale bar, 200 μm; **(B)** Percentages of cleavage and blastocyst rates after maternal prepregnancy 5-HT exposure; **(C)** Images of the uterus with implantation sites at E13.5 after maternal prepregnancy 5-HT exposure. Red arrow, a small implantation site being absorbed; **(D)** The percentage of embryo resorptions at E13.5 in individual mice; and **(E)** Weights of 2-week-old offspring after maternal prepregnancy 5-HT exposure. *n* = 6, ^*^p < 0.05, and ^**^p < 0.01.

To explore whether the maternal effects of 5-HT were transmitted to the embryo and fetus, we first examined the influence of 5-HT on preimplantation embryo development. The results showed that there were no significant differences in the cleavage and blastocyst rates between the two groups (Figures 4C,D), although maternal weight and placental development in the 5-HT group were obviously suppressed compared with those in the control group.

### Effects of Maternal Prepregnancy 5-HT Exposure on the Fetus

Furthermore, we investigated the effect of maternal prepregnancy 5-HT exposure on the fetus at E13.5. Notably, mice derived from 5-HT exposure had resorbed implantation sites (Figure 4A), and the average resorption rate was markedly higher than that in the control group (Figure 4B; *p* < 0.01), which was inconsistent with preimplantation embryos. In detail, mice in the 5-HT group had resorbed implantation sites that were small but still partially pinkish and had degraded placental tissues, while fetal tissues were completely absorbed (Figures 3C, 4A). These data demonstrated the negative effect of 5-HT exposure on the development of offspring in mice.

### Effects of Maternal Prepregnancy 5-HT Exposure on Offspring Growth

To better ascertain whether maternal prepregnancy 5-HT exposure regulates the development of offspring, we measured the weights of 2-week-old offspring derived from natural delivery. Statistical analysis revealed that 5-HT exposure caused a significant reduction in the weights of 2-week-old offspring (Figure 4E; *p* < 0.05). These results suggested that maternal prepregnancy 5-HT exposure did not affect preimplantation embryo development, while there were obvious negative effects on the development of the fetus and offspring.

## Discussion

It has been claimed that the environment experienced by the parents affects the offspring who have never experienced the environment (Dietz et al., 2011; Seong et al., 2011; Rando, 2012). For example, exposure to environmental challenges, such as stress, toxins, or an unhealthy diet, can affect the development of the parents (Han et al., 2018). The changes could then be passed on to offspring and could make them more susceptible to illness (Han et al., 2018). Therefore, the change in maternal developmental level is closely related to the developmental ability of the offspring. When the mother takes an overdose of antidepressants, it can even cause 5-HT syndrome, resulting in an increase in 5-HT in the body (Bartlett, 2017). However, whether the increase in 5-HT and even 5-HT syndrome in mothers affects the development of offspring has not yet been studied. Thus, we aimed to understand the potential effects of maternal 5-HT levels on the mother and fetus. In the present study, we first found that the offspring of mothers injected with 5-HT had lower body weight at 2 weeks, which indicates that the mother having 5-HT syndrome before pregnancy may affect the development of the offspring. Previous research mainly focused on obesity, depression, and other diseases of the mother during pregnancy that would affect the development of offspring. Therefore, the result that the prepregnancy changes in the mother affected the development of the offspring in this study is enough to attract more research attention.

In the gastrointestinal tract, 5-HT is a key signaling molecule involved in intestinal secretion, sensation, and peristalsis (Bulbring and Crema, 1958; Gershon, 1999; Shajib and Khan, 2015). It has been demonstrated that 5-HT is involved in mediating interdigestive contractions of the small intestine in rats (Pineiro-Carrero et al., 1991). 5-HT3 and 5-HT4 receptors play essential roles in regulating intestinal interdigestive contractions (Taniguchi et al., 2008). In the present study, we found that intraperitoneal injection of 5-HT could prevent the increase in maternal weight and reduce hormone levels before and during pregnancy in female mice, which was consistent with a previous study in rats (Edwards, 1995). Simansky reported that neural 5-HT could inhibit food intake and tends to reduce body weight gain and even increase energy expenditure (Simansky, 1996). Therefore, we speculate that 5-HT injection affects the contraction and peristalsis of the intestines, and reduces nutrient intake; on the other hand, 5-HT injection inhibits the appetite of mice and reduces food intake, which ultimately causes slower weight gain. We also noticed that there was a reduction in placental weight caused by 5-HT exposure. When the development of the placenta is impaired, the nutrients, hormones, and growth factors that were necessary for the development of the embryo and fetus cannot be passed from the mother, contributing to the abnormal growth and development of offspring. This may explain the increasing fetal absorption rate in the 5-HT group, as well as the reduced body weight of offspring.

Ovarian hormones are known to perform important roles related to various neurotransmitters, such as 5-HT, the noradrenergic system, and glutamate (Paredes et al., 2019). For example, sex hormones affect the synthesis, reuptake, and degradation of the serotoninergic system. Moreover, previous studies have reported that estrogens could regulate the serotonergic system to protect against 5-HT-related diseases. However, in turn, estrogen synthesis was affected to restore homeostasis by 5-HT (Hudon Thibeault et al., 2019). In the present study, we found that 5-HT exposure could reduce maternal estrogen and progesterone levels, which was likely based on the negative feedback effect of 5-HT on hormone synthesis. Low concentrations of estrone-sulfate and estradiol-17β were found in cows that gave birth to stillborn calves, which emphasizes the importance of maintaining proper estrogen concentrations during pregnancy (Kindahl et al., 2004; Nguyen et al., 2012). In primate pregnancy, estrogen deprivation leads to insulin resistance in offspring (Maniu et al., 2016).

5-HT has an essential role in the regulation of embryo development, but the explicit effects remain controversial (Chebotar et al., 1995; Sakharova et al., 1997; Vesela et al., 2003; Il'kova et al., 2004). Our results showed that maternal prepregnancy 5-HT exposure did not affect the cleavage and blastocyst rate in preimplantation embryos. However, much evidence has shown that maternal changes in the environment, nutrition, and endocrine stress during pregnancy can interfere with epigenetic modifications, which further affects gene expression and the growth and development of offspring (Marco et al., 2016; Wang et al., 2016a). For instance, epigenetic changes have been observed in the offspring of the Cohen strain of diabetic rats and human placenta (Ornoy et al., 2015; Petropoulos et al., 2015). In mice, maternal high-fat intake altered mRNA m6A modifications in adipose and skeletal muscle tissues in offspring (Li et al., 2016). The effects of these epigenetic changes may appear in the pro-implantation stage and after birth, rather than in the preimplantation embryo. Further exploration is needed to determine whether maternal 5-HT exposure contributes to the changes in epigenetic modification.

A previous study confirmed that both 5-HT and estrogen are important regulators of the immune system (Shajib and Khan, 2015). In the 1980s, 5-HT was identified as an immunomodulator because of its ability to stimulate or inhibit inflammation (Cloez-Tayarani et al., 2003). In mice, 5-HT administration increased the release of proinflammatory cytokines in macrophages in dextran sodium sulfate (DSS)-induced colitis (Khan and Ghia, 2010). 5-HT can promote the recruitment of mast cells, eosinophils, DCs, and neutrophils in acute inflammation (Boehme et al., 2004). In addition, there is a strong connection between estrogen levels and inflammation (Au et al., 2016). Women who are ovariectomized as well as those with natural menopause exhibit systemic inflammation (Cioffi et al., 2002; Abu-Taha et al., 2009). In the present study, we found that maternal prepregnancy 5-HT exposure caused inflammation in the placenta and increased the expression of proinflammatory markers, which might result from changes in 5-HT and estrogen levels. Therefore, placental inflammation may be directly caused by maternal 5-HT exposure, or 5-HT may indirectly promote inflammation by reducing estrogen levels. Further study should be performed examining the underlying mechanism of this phenomenon.

Maternal inflammation during pregnancy can affect the development of the fetus by interrupting murine intestinal development, influencing placental function, and increasing the risk of neurodevelopmental disorders (Romero et al., 2007; Elgin et al., 2019). In the present study, prepregnancy 5-HT exposure caused inflammation in the placenta and reduced the weights of 2-week-old fetuses. The placenta is an important communication organ between the mother and fetus. Inflammation of the placenta may affect nutrient transfer from the mother to the fetus, which further leads to the abnormal development of the fetus. It ultimately impacts offspring development and causes growth retardation and slow weight gain in offspring.

In summary, our research showed that maternal prepregnancy 5-HT exposure could affect offspring development by reducing maternal body weight, suppressing estrogen and progesterone secretion, and causing placental inflammation. This study provides a basis for the relationship between 5-HT and intergenerational inheritance and opens up new perspectives for future research directions.

## Data Availability Statement

The original contributions presented in the study are included in the article/supplementary material, further inquiries can be directed to the corresponding author.

## Ethics Statement

The animal study was reviewed and approved by The Animal Care and Use Committee of Jilin University.

## Author Contributions

BT designed and coordinated the study. YH performed the embryo culture and ELISA and wrote the manuscript. YH, MZ, and JD collected tissues and weighed. LL, JD, and HC performed the qPCR. SZ, YZ, XA, and QL provided the technical assistance and contributed to the data analysis. ZL and XZ revised the manuscript. All authors contributed to the article and approved the submitted version.

## Funding

The study was funded by the National Natural Science Foundation of China (no. 31472093; 31872434; and 32172803), the National Key R&D Program of China (no. 2017YFA0104400), and the Scientific Research Project of Education Department of Jilin Province (JJKH20211136KJ).

## Conflict of Interest

The authors declare that the research was conducted in the absence of any commercial or financial relationships that could be construed as a potential conflict of interest.

## Publisher’s Note

All claims expressed in this article are solely those of the authors and do not necessarily represent those of their affiliated organizations, or those of the publisher, the editors and the reviewers. Any product that may be evaluated in this article, or claim that may be made by its manufacturer, is not guaranteed or endorsed by the publisher.
